# Theoretical Study of the Influence of K20N Glycosylation on the Dynamic Behavior of Im7 Protein

**DOI:** 10.3390/molecules30193939

**Published:** 2025-10-01

**Authors:** Jianqiang Wang, Panpan Wang, Guojie Cheng, Dawei Zhang

**Affiliations:** School of Physics and Engineering, Henan University of Science and Technology, Luoyang 471023, China; 230320090792@stu.haust.edu.cn (J.W.); ppw2750@163.com (P.W.); gjcheng1@163.com (G.C.)

**Keywords:** N-linked glycosylation, Im7 protein, urea solution

## Abstract

This study employed molecular dynamics simulations to investigate the impact of N-linked glycosylation (GlcNAc_2_) at the K20N position on the structural dynamics and stability of the bacterial immunity protein Im7. Simulations were conducted in both aqueous and 2 M urea denaturing environments. The simulation results in aqueous solution indicate that glycosylation has only a minor effect on the protein, consistent with expectations. In contrast, simulations in urea reveal that K20N glycosylation significantly destabilizes Im7. Analyses of RMSD, native contacts, SASA, RMSF, correlation matrix, PCA, helical content and hydrophobic centroid distance consistently demonstrate that K20N glycosylation increases the flexibility of Helix I and Helix II and weakens the concerted motion among helical domains (particularly between Helix I and Helix II/IV). The destabilizing effect of K20N glycosylation on Im7 originates in Helix I (where glycan attaches) and propagates to Helix II and the loop region connecting Helix I and Helix II. The instability of Helix II is closely associated with the disruption of hydrophobic interactions within the hydrophobic core. These findings provide new insights into the relationship between site-specific glycosylation and protein stability.

## 1. Introduction

Protein glycosylation, as an important post-translational modification, plays a critical regulatory role in the structural stability, dynamic behavior, and biological functions of proteins [1,2,3,4,5,6]. Currently, the most commonly used methods for resolving the three-dimensional structures of protein molecules are X-ray crystallography and nuclear magnetic resonance (NMR) spectroscopy. Both techniques analyze protein structures in an aqueous solution, which differs from the crowded and complex intracellular environment where proteins are actually functional. In cells, specific amino acids on protein surface are modified by sugar molecules (glycans), so the protein structures resolved under aqueous conditions may deviate from their native cellular forms. In recent years, the use of artificial intelligence (AI) methods to predict protein three-dimensional structures has become a prominent research focus. However, when predicting glycosylated protein structures, the scarcity of experimental data in this area increases the difficulty of AI predictions. Therefore, there is an urgent need to expand the database on how glycosylation influences protein structural stability.

Protein glycosylation can be classified into different types based on the connection between sugar and side chains of amino acids. The two most common types are N-linked [7] and O-linked [8] glycosylation. N-linked glycosylation occurs when a glycan attaches to the amide group of asparagine (Asn) within the conserved sequence N-X-S/T (where X is any amino acid other than proline) [9,10]. This is the predominant form of glycosylation in eukaryotic cells. O-linked glycosylation is the process of attaching glycans to the hydroxyl groups of serine (Ser) and threonine (Thr) [11]. In contrast to N-linked glycosylation, O-linked glycosylation can occur in multiple cellular compartments, including the endoplasmic reticulum, Golgi apparatus, cytoplasm or nucleus.

**Figure 1:**
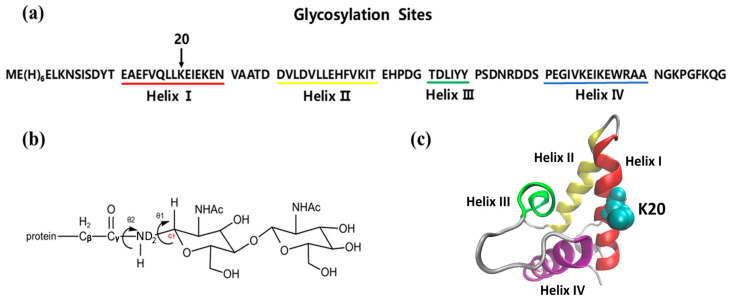
(**a**) Amino acid sequence of Im7 protein showing Helices I-IV and K20N glycosylation site; (**b**) structure of the oligosaccharide (GlcNAc-GlcNAc) attached to Im7; (**c**) structure of Im7 protein.

Recent studies have shown that glycosylation can alter the stability of proteins, revealing its importance in biological research [12,13,14]. However, the experimental attachment of glycans to proteins remains technically challenging, which has limited progress in glycosylation research. Among the limited studies in this field, we selected the bacterial immunity protein Im7 [15,16,17,18,19], which has been extensively studied, as the focus of the work. The Im7 protein, composed of 87 amino acids, has a molecular weight of about 10.3 kDa. It adopts a compact four-helix bundle structure without disulfide bonds, as illustrated in Figure 1. Its small size facilitates the synthesis and handling of glycoconjugates, making it an excellent model system for investigating how glycosylation influences protein stability.

In this study, we investigated the structural consequences of glycosylation using Im7 as a model system. Based on its crystal structure, N-linked glycosylation modification (GlcNAc-GlcNAc) at the K20N position was introduced via in silico modeling. The site was chosen because it is located in the middle of α-helix on the protein surface, away from the hydrophobic core, making it an ideal location to assess how glycosylation perturbs a stable secondary structural element [19]. To validate our approach, the glycosylated Im7 was first simulated in aqueous solution. Although the results confirmed the feasibility of our model, only minor structural alterations were observed. To amplify the effects of glycosylation, simulations were subsequently carried out in a 2 M urea denaturing environment. It is worth to note that the use of 2 M urea solution in our study was not intended to mimic a physiological condition for Im7. Instead, it was adopted to match the experimental setup in the in vitro unfolding study performed by Barbara Imperiali and colleagues [19] to allow for a meaningful comparison between our simulation results and their experimental findings. The obtained trajectories were then analyzed to evaluate how K20N glycosylation affects the stability of Im7.

## 2. Simulation Details

### 2.1. Carbohydrate Building

The disaccharide (GlcNAc-GlcNAc, GlcNAc:β-1,4-N-acetylglucosamine) was named according to the residue nomenclature rules of the GLYCAM force field. The N-acetylglucosamine residues were labeled 4YB and 0YB, where ‘4’ indicates a 1→4 glycosidic linkage (C4 of one sugar bound to C1 of the next), ‘0’ denotes a terminal sugar with no further connections, ‘Y’ represents N-acetylglucosamine, and ‘B’ specifies a β-configured pyranose ring in a chair conformation (Figure 1b). The oligosaccharide was constructed using the LEaP program with the GLYCAM_06j-1 force field, which is compatible with both standalone carbohydrate modeling and combined use with the AMBER ff14SB force field.

### 2.2. Glycoprotein Construction

The X-ray crystal structure of wild-type Im7 was obtained from the RCSB Protein Data Bank (PDB) with a PDB id of 1AYI [20] and was used as a template in the modeling of non-glycosylated and glycosylated Im7. All non-standard atoms, including ligands, non-crystallographic water molecules, and uncoordinated metal ions, included in the X-ray structure were removed prior to the modeling. Following previous work by Barbara Imperiali et al., a K20N mutation was introduced to create an N-linked glycosylation site at position 20 (Figure 1a) [19]. Additionally, residue 29 was mutated to cysteine to enable chemo-selective ligation for subsequent glycopeptide synthesis via solid-phase peptide synthesis (SPPS) [21,22]. The mutation step is simplified with the aid of the Maestro program to place the side chains of Asn20 and Cys29 using standard rotamer libraries [23]. The resulting mutant structure was then processed with the Protein Preparation Wizard to perform local energy minimization [23], relieving any potential steric clashes introduced by the mutations. This optimized structure served as the starting point for subsequent glycosylation. The glycosylated structure was generated using the LEaP program with combined AMBER ff14SB (protein) and GLYCAM_06j-1 (carbohydrate) force fields. The resulting glycosidic linkage is characterized by two key torsion angles: θ1 (H1-C1-ND2-HD2) and θ2 (C1-ND2-Cγ-Cβ). As shown in Figure 1b and consistent with NMR studies by Yan Xue et al., the linkage adopts a stable θ1-anti/θ2-trans conformation [24]. Combined with the Φ (ND2-Cγ-Cβ-Cα) angle, this set of three torsions comprehensively characterizes the Asn-glycan linkage and describes the spatial orientation of both the Asn20 and the glycan relative to the protein. The time evolution of the three torsion angles is provided in Supporting Figure S1. Based on this, all simulations were conducted to compare the glycosylated Im7 (K20N*/A29C) against the non-glycosylated Im7 (K20N/A29C) control, ensuring that the observed differences are attributable solely to the presence of the glycan.

### 2.3. Urea Solvent Box Preparation

Based on experimental results by Barbara Imperiali et al., the Im7 structure undergoes partial unfolding in 2 M urea solution [19]. To maintain consistency with experimental conditions, simulations of both non-glycosylated and glycosylated Im7 were conducted in an explicit 2 M urea solvent environment. The urea box was prepared by diluting an 8 M urea box from AmberTools16 with TIP3P water molecules to achieve an approximate concentration of 2 M. This resulted in a rectangular box (66 Å × 63 Å × 69 Å) containing 330 urea molecules and 6355 water molecules [25,26,27,28]. The density profile and total potential energy of the system were monitored to validate the stability and proper concentration of the final urea–water mixture (Figure S2 in Supporting Information). After preparing the urea solution, energy minimization was conducted using the steepest descent method for 10,000 steps, followed by 10,000 steps of conjugate gradient minimization. The system was then heated from 0 K to 300 K over 100 ps using a Langevin thermostat (collision frequency = 4 ps^−1^) in the NVT ensemble [29,30]. Subsequently, a 2 ns molecular dynamics (MD) simulation was conducted in the NPT ensemble to ensure proper urea–water mixing at constant temperature (300 K) and pressure. All simulations employed the Generalized Amber Force Field (GAFF) [31], with urea parameters and charges taken from Özpinar et al. [32].

### 2.4. Simulation Protocol

After constructing the equilibrated 2 M urea box, both the glycosylated Im7 protein (with K20N*/A29C mutations) and non-glycosylated Im7 protein (with K20N/A29C mutations) were separately solvated in the urea–water mixture within an octahedral box, maintaining a minimum distance of 15 Å between the protein and the box edge. For each system, Na^+^ ions were added using the LEaP program to neutralize system charge [25]. Prior to the productive runs mimicking the experimental unfolding of glycosylated Im7 performed by Barbara Imperiali et al. [19], the solvent surrounding the protein was energy-minimized in two stages: (1) 10,000 steps of steepest descent minimization, followed by (2) 10,000 steps of conjugate gradient minimization. The complete protein–solvent system was then minimized with 5000 steps of steepest descent, followed by conjugate gradient minimization until an energy gradient convergence criterion of ≤0.01 kcal/mol/Å was reached. The system was subsequently heated from 0 K to 300 K over 100 ps in the NVT ensemble using a Langevin thermostat (collision frequency = 4 ps^−1^) [29,30]. Productive runs were performed for both non-glycosylated and glycosylated Im7 in the NPT ensemble. All simulations used a 2 fs timestep, with long-range electrostatics handled by the particle mesh Ewald method using a 12 Å cutoff and hydrogen-involved covalent bonds constrained via the SHAKE algorithm [33,34]. The force fields used were AMBER ff14SB for the protein, GLYCAM_06j-1 for the carbohydrate, and GAFF for the urea solution [31,35,36]. It is worth noting that the primary goal of these simulations was not to capture the equilibrium fluctuations in a native state but to probe the systematic destabilizing effect of glycosylation under the accelerated unfolding conditions (2 M urea).

## 3. Results and Discussions

Initial simulations compared the dynamic behaviors of non-glycosylated and K20N-glycosylated Im7 proteins in aqueous solution. The results (Figure S3 in Supporting Information) agreed with experimental observations, although the differences were relatively minor. This agreement validated the simulation approach. Subsequent simulations examined both proteins in urea–water solution. Urea, a well-known protein denaturant, promotes protein unfolding. Simulating glycosylated protein in urea solution amplifies glycosylation-induced differences in dynamic behavior and allows for the observation of glycosylation-induced destabilization within a relatively shorter timeframe while also maintaining consistency with the experimental conditions used by Barbara Imperiali et al. [19].

**Figure 2:**
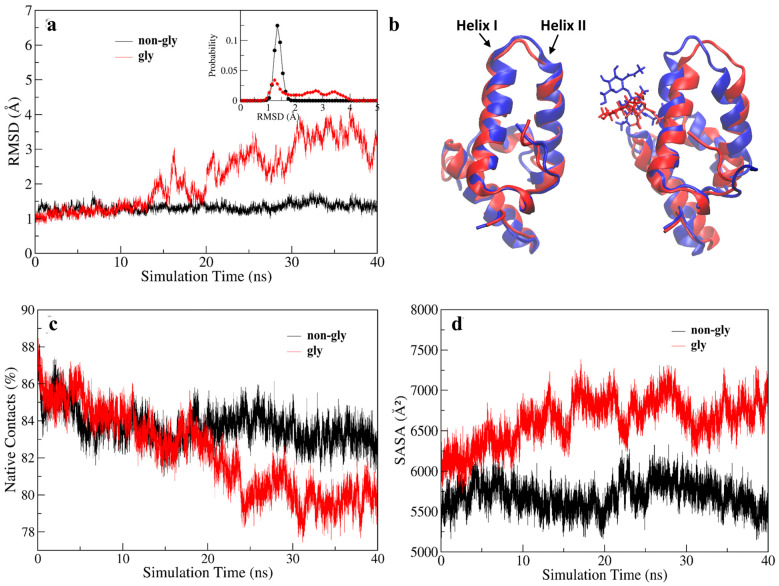
(**a**) Variation in backbone (N-CA-C-O) RMSD of non-glycosylated and glycosylated Im7 with RMSD distribution inserted; (**b**) The first and last frame from the MD trajectories of both non-glycosylated and glycosylated Im7 simulated in the 2 M urea environment were superimposed to visualize the conformational changes over the 40 ns simulation; (**c**) variation in native contacts over time for non-glycosylated and glycosylated Im7; (**d**) SASA over time for non-glycosylated and glycosylated Im7.

**Root-mean-square deviation (RMSD):** RMSD quantifies the deviation of a structure from a reference conformation during simulation [37]. The higher the RMSD value, the greater the structural deviation, and it can also serve as an indicator of system equilibrium. To examine conformational changes, RMSD values were calculated using the energy-minimized structure as the reference. The glycosylated Im7 exhibited a significant increase in backbone (N-CA-C-O) RMSD after 10 ns, as shown in Figure 2a. This trend was consistently observed in analyses based solely on the Cα atom and on all sidechain atoms (Figure S4 in Supporting Information). The probability distribution shows a narrow peak for non-glycosylated Im7 and a broad peak for glycosylated Im7, indicating greater structural diversity after glycosylation. The first and last frames from the MD trajectories of both non-glycosylated and glycosylated Im7 were extracted and superimposed to visualize the conformational changes over the 40 ns simulation. As shown in Figure 2b, the last frame of glycosylated Im7 shows significant deviation from its starting structure, particularly in the region of Helix I, Helix II, and the connecting loop. In contrast, non-glycosylated Im7 remains much more compact and retains its native four-helix bundle fold throughout the simulation, showing minimal deviation from its initial structure. These results are consistent with the experimental findings of Barbara Imperiali et al. [19], suggesting that K20N glycosylation reduces the stability of Im7. Further analytical methods will be used to explore the molecular mechanism of this glycosylation-induced destabilization.

**Native contacts:** Native contacts refer to the non-covalent interactions between amino acid residues that are adjacent in three-dimensional space but distant in the primary sequence. The fraction of native contacts is defined using a 5.0 Å cutoff in the reference (energy-minimized structure), with Q (t) calculated via a simple cutoff scheme, and its preservation provides a quantitative measure of protein stability. In molecular dynamics simulations, less stable proteins lose native contacts more rapidly. Figure 2c shows that in the first half of the simulation, non-glycosylated and glycosylated Im7 have almost the same native contact profiles, indicating minimal initial influence of glycosylation. After 20 ns, however, significant differences emerged. Compared to non-glycosylated Im7, the K20N-glycosylated Im7 showed significantly faster native contact loss. This suggests that K20N glycosylation may induce changes in inter-residue interactions, promote protein conformational rearrangement, and thereby accelerate protein instability.

**Solvent-accessible surface area (SASA):** In addition to native contacts, hydrophobic interactions play a key role in stabilizing globular proteins. Hydrophobic interactions between non-polar amino acids shield hydrophobic cores from the aqueous environment, maintaining structural stability [38]. In experimental studies, UV fluorescence spectroscopy is usually used to monitor hydration and collapse of hydrophobic cores during the start of protein unfolding [39,40,41,42,43,44]. This spectral technique takes advantage of the intrinsic fluorescence properties of proteins to sensitively detect changes in the tertiary structure of proteins via variations in the solvent accessibility of hydrophobic regions [39,40,41,42]. During denaturation, unfolding of proteins inevitably leads to a loss of hydrophobic interactions, exposing the hydrophobic core to solvent. In this work, the solvent-accessible surface area was computed using the surf command in CPPTRAJ with a probe radius of 1.4 Å. Figure 2d shows that the SASA value of glycosylated Im7 gradually increases, reflecting structural rearrangement that exposes buried residues. This SASA analysis suggests that K20N glycosylation disrupts the hydrophobic core, potentially explaining its destabilization effects.

**Figure 3:**
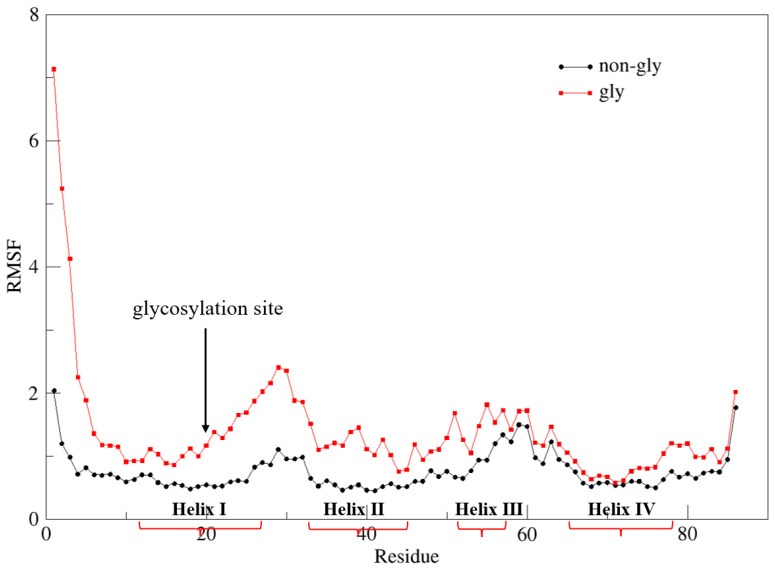
Backbone RMSF curves for non-glycosylated and glycosylated Im7 proteins.

**Root-mean-square fluctuation (RMSF).** While previous analytical methods focused on global structural properties, residue-level analyses clarify the intrinsic destabilization of Im7 caused by K20N glycosylation. The root-mean-square fluctuation (RMSF) quantifies the displacement of individual amino acid residues relative to the average position during simulation, providing a measure of local structural flexibility. To examine residue-specific flexibility, the backbone RMSF analysis was carried out to reveal glycosylation-induced changes in protein dynamics. As shown in Figure 3, a significant increase in fluctuation is observed near the glycosylation site (residue 20), particularly in Helix I, Helix II, and their connecting loop, with the amplitude decreasing as the distance from the modification site increases. Consistent with this trend, RMSF analyses based solely on the Cα atom and on sidechain atoms, respectively, yielded similar patterns (Figure S5 in Supporting Information). Since the K20N glycosylation site is located in the middle of Helix I, theoretically, glycosylation has the greatest impact on this domain. However, RMSF analysis shows that this influence also propagated to Helix II and the connecting loop region.

**Figure 4:**
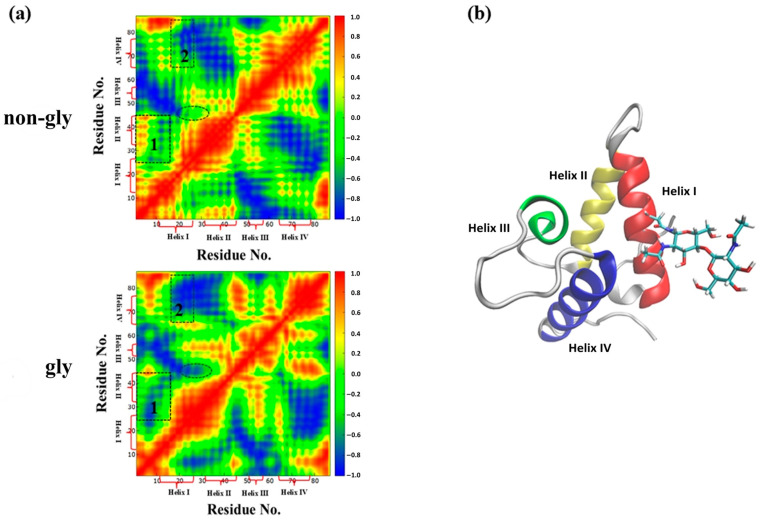
(**a**) Correlation map for non-glycosylated and glycosylated Im7; (**b**) structure of the K20N-glycosylated Im7.

**Correlation map:** By analyzing trajectories of non-glycosylated and glycosylated Im7, correlation matrices characterizing dynamic correlations between helical domains were obtained. This two-dimensional representation method effectively captures motion correlations between protein residues [45,46,47]. As shown in Figure 4, red regions indicate positively correlated motion and blue regions indicate negatively correlated motion. These motion correlations may arise from direct contact or long-range interactions between domains. Comparative analysis in Figure 4 reveals two regions involving Helix I that exhibit significant glycosylation-induced changes: Region 1 (correlation between Helix I and Helix II) and Region 2 (correlation between Helix I and Helix IV). K20N glycosylation expanded the blue negatively correlated regions and reduced the red positively correlated regions, suggesting weakened dynamic coupling between helices enclosing the hydrophobic core, particularly between Helix I and Helix II, as well as between Helix I and Helix IV. Reduced motion correlation indicates weakened concerted movement among hydrophobic residues, which may promote the exposure of the hydrophobic core to solvent and reduce stability.

**Figure 5:**
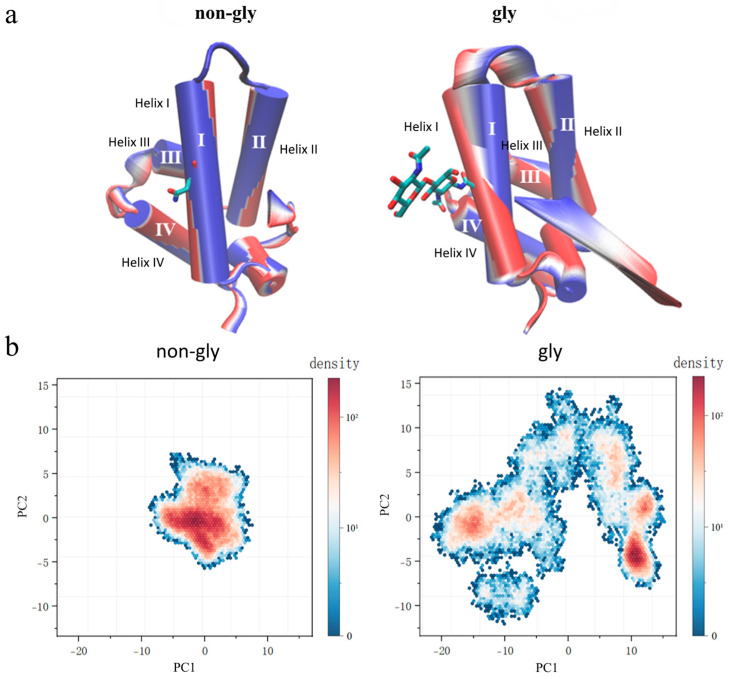
(**a**) Projection of the motion along the first eigenvector for non-glycosylated and glycosylated Im7 obtained through PCA. The color transition from the first to the last frame goes from red to white to blue. (**b**) 2D projection of the first two principal components (PC1 vs. PC2) calculated for non-glycosylated and glycosylated Im7.

**Principal component analysis (PCA):** MD simulations in urea solution revealed key unfolding events in Im7, characterized by enhanced structural fluctuations in helical domains (particularly Helix I and Helix II) and the connecting loop, as well as destruction of the hydrophobic core. To further confirm these analyses, PCA was applied to visually characterize the degree of fluctuations in Helix I, Helix II, and the connecting loop, aiming to link conformational changes to the stability of glycosylated Im7 versus non-glycosylated Im7. As a well-established technique, PCA reduces the complex 3N-dimensional conformation space (where N represents the number of atoms) to essential modes of motion [48,49,50]. Trajectories of both systems were analyzed using PCA, and key modes related to unfolding were visualized using the interactive essential dynamics (IED) tool in VMD [51,52]. As shown in Figure 5a, glycosylated Im7 shows a much wider amplitude of the dominant collective motion captured by the first principal component (PC1) and exhibits pronounced displacement in Helix I, Helix II, and the connecting loop compared to non-glycosylated protein. Significant twisting and swinging motions of Helix I and Helix II in glycosylated Im7 may exacerbate disruption of the hydrophobic core, thereby accelerating unfolding. Two-dimensional projection of PC1 vs. PC2 quantifies the conformational sampling of proteins in the space defined by the two most significant motions. As shown in Figure 5b, non-glycosylated Im7 is located in a narrow and clearly defined region within the conformational space formed by the first two principal components, indicating that it has restricted motion within the range corresponding to the native structure. In contrast, the glycosylated variant explores a much broader conformational space, which can be demonstrated by the extensive dispersion of the projected distribution. This significant expansion of conformation sampling suggests that glycosylation leads to the instability of the structure. The observed increase in flexibility, especially in the helical segments near the modified site, is consistent with the destabilization of the native structure. These principal component analysis results directly link glycosylation to the fundamental reshaping of the protein energy landscape, providing a dynamic perspective that is consistent with the instability observed in other structural indicators.

**Figure 6:**
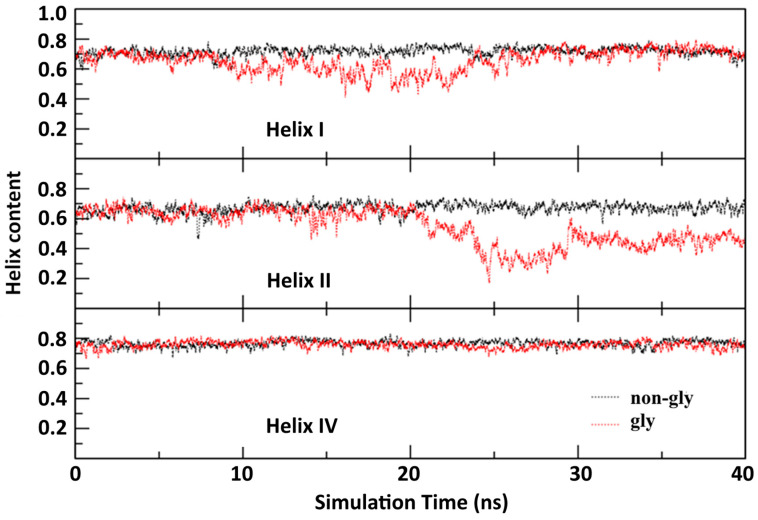
Variation in helical contents in both non-glycosylated and glycosylated Im7 proteins with simulation time.

**Helix content:** The location of the K20N glycosylation site in the middle of Helix I suggests that this domain may experience the most direct effect. Consistent with this expectation, the RMSF, correlation matrix, and PCA jointly demonstrated dynamic changes in Helix I and adjacent Helix II upon glycosylation. To evaluate the impact of glycosylation on helical stability, we calculated the helical content over time using the DSSP algorithm, implemented in CPPTRAJ for all helices except Helix III (3_10_ helix), as helical content is a reliable indicator of helical stability (higher values indicate greater stability). As shown in Figure 6, the helical content of Helix I of glycosylated Im7 decreased in the first half of the simulation but recovered later. In contrast, Helix II was stable initially but showed significant loss of helical content in the second half. Helix IV remained stable throughout. These findings clarify the structural mechanism by which glycosylation in Helix I propagates destabilization, particularly affecting adjacent Helix II while leaving other helices largely unchanged.

**Figure 7:**
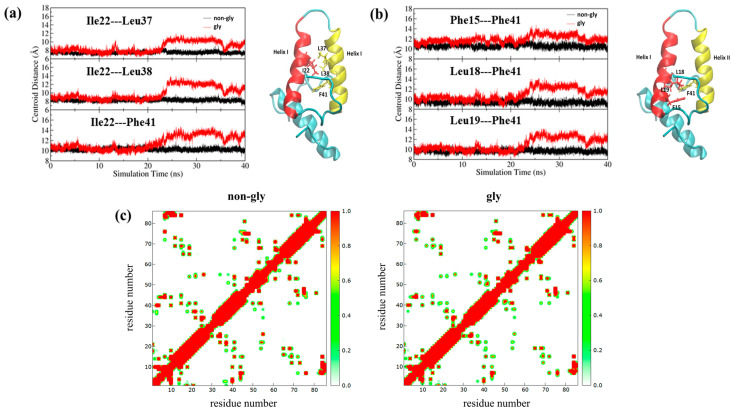
(**a**) Variation in centroid distances between Ile22 in Helix I and Leu37, Leu38, and Phe41 in Helix II over simulation time with structure diagram of Im7 showing the four hydrophobic residues mentioned above; (**b**) variation in centroid distances between Phe41 in Helix II and Phe15, Leu18, and Leu19 in Helix I over simulation time with structure diagram of Im7 showing the four hydrophobic residues mentioned above; (**c**) comparison of contact map for non-glycosylated and glycosylated Im7.

**Centroid distance:** The formation of tightly packed hydrophobic cores is essential for globular proteins to maintain stability, natural conformation, and function. Like other globular proteins, the stability of Im7 heavily depends on the integrity of its hydrophobic core. To evaluate the direct effect of K20N glycosylation on this core, centroid distances between key hydrophobic residues in Helix I and Helix II were analyzed. Figure 7 shows the dynamic changes in centroid distances between Ile22 (Helix I) and Leu37/Leu38/Phe41 (Helix II) and between Phe41 (Helix II) and Phe15/Leu18/Leu19 (Helix I) with simulation time. As can be seen, all measured distances increased consistently in the second half of the simulation, indicating loss of structural integrity in the hydrophobic core. This geometric observation is quantitatively corroborated by the contact map in Figure 7c. The specific regions corresponding to the inter-helical contacts between Helix I and Helix II show a striking color shift from red (indicating a high percentage of simulation time in close contact) in non-glycosylated Im7 to green or even disappearance (indicating a low percentage of contact time) in the glycosylated variant. This visual change quantitatively confirms the drastic reduction in close-range interactions between these helices. Combined with the helical content analysis, these results suggest that the instability of Helix II is related to increased centroid distances between these hydrophobic residues.

## 4. Conclusions

Molecular dynamics simulations of the Im7 protein in a 2 M urea denaturation environment revealed that N-linked disaccharide (GlcNAc-GlcNAc) modification at the K20N site significantly destabilizes Im7. Comprehensive analyses indicate that glycosylation at the mid-point of Helix I increases the flexibility of this helical domain, and this effect propagates to adjacent regions, particularly Helix II and the loop connecting Helices I and II. The increased flexibility of the helical domains disrupts dynamic coupling between helices (particularly Helix I-II and Helix I-IV) and weakens the structural integrity of the hydrophobic core, as evidenced by increased SASA and hydrophobic centroid distance. These changes accelerate the loss of native contacts and reduction in helical content, ultimately promoting the unfolding of Im7. These findings establish a clear relationship between site-specific glycosylation and alterations in protein stability, providing new molecular-level insights into how this post-translational modification influences protein dynamics.

## Data Availability

The data that support the findings of this study are not publicly available because the author’s university restricts peripheral access but it can be made available by the corresponding author upon reasonable request.

## Supplementary Materials

The following supporting information can be downloaded at: https://www.mdpi.com/article/10.3390/molecules30193939/s1, Figure S1. Time evolution and distribution of torsion angle θ1, θ2 and Φ. Figure S2. Density (a) and total potential energy (b) profiles of the urea-water mixture. Figure S3. Simulations in aqueous solution with backbone RMSD (a) and SASA (b) for non-glycosylated and glycosylated Im7 respectively. Figure S4. Variation in RMSD based on Cα and sidechain atoms for non-glycosylated and glycosylated Im7. Figure S5. Cα-RMSF and sidechain-RMSF for non-glycosylated and glycosylated Im7.
