# Supplementary material for: Theoretical Study of the Influence of K20N Glycosylation on the Dynamic Behavior of Im7 Protein
Source: Molecules. 2025 Oct 1;30(19):3939. doi: 10.3390/molecules30193939 (PMC12525938; doi:10.3390/molecules30193939)
Supplement: Supplementary file 1 [file molecules-30-03939-s001.zip › molecules-3889162-supplementary.pdf]

Supporting Information

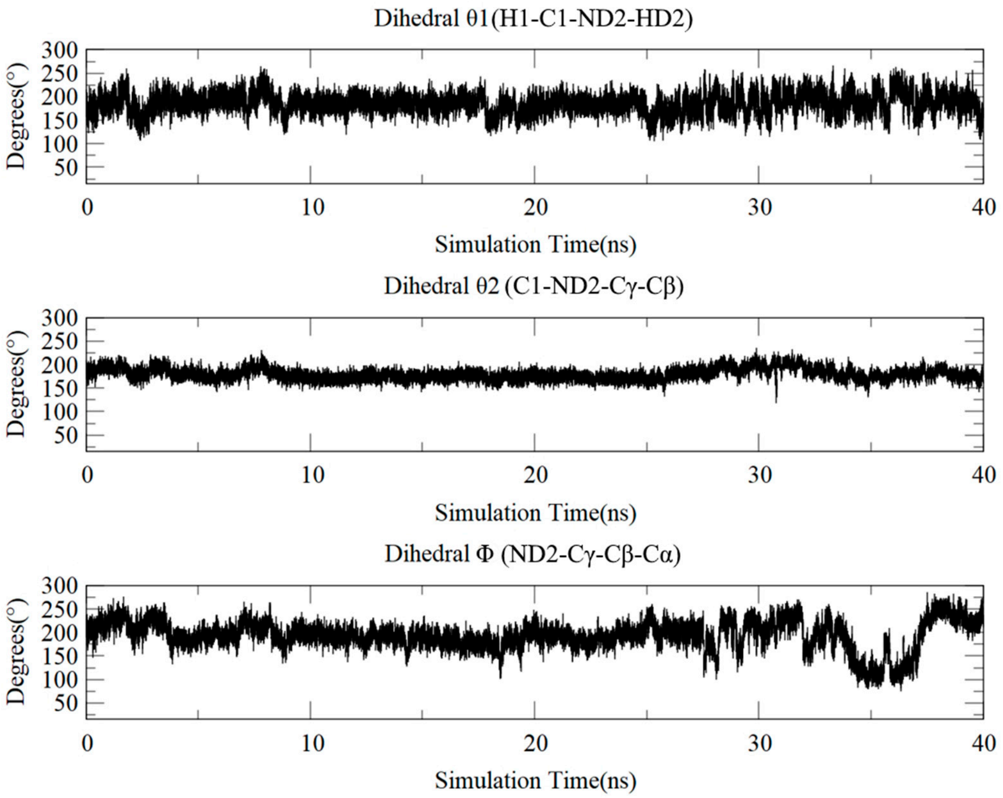

Figure S1. Time evolution and distribution of torsion angle θ1, θ2 and Φ.

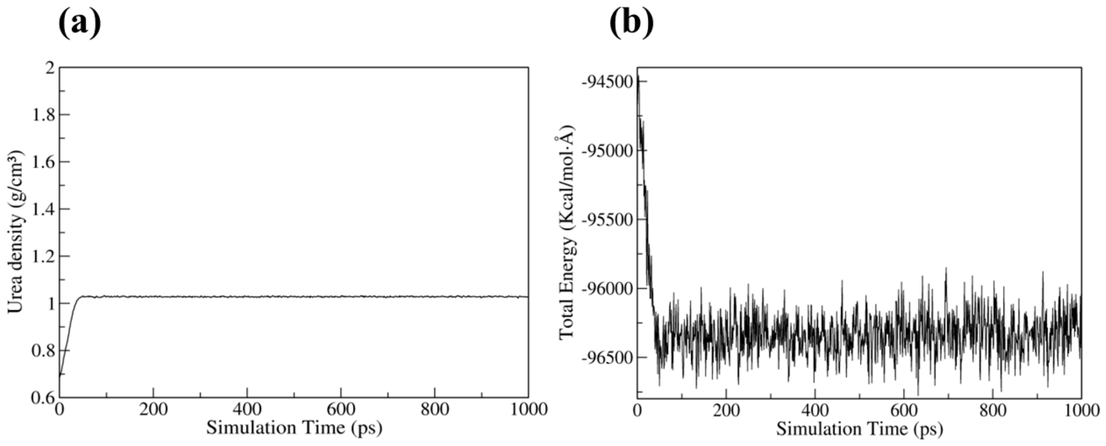

Figure S2. Density (a) and total potential energy (b) profiles of the urea-water mixture.

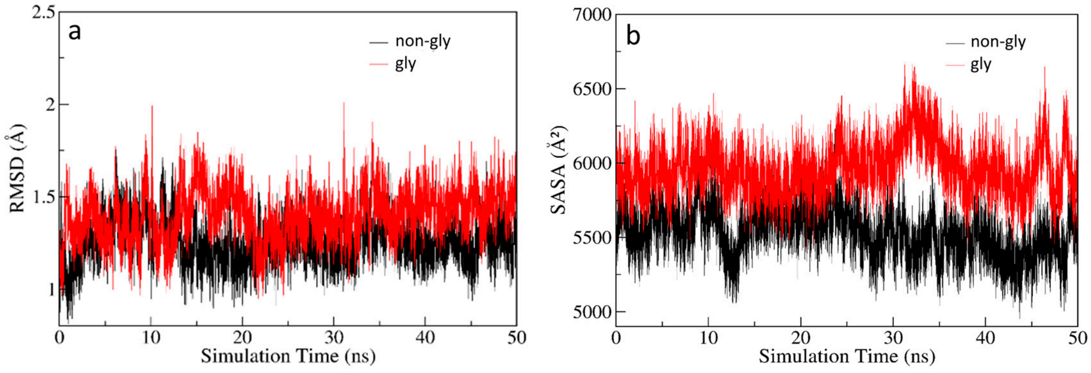

Figure S3. Simulations in aqueous solution with backbone RMSD (a) and SASA (b) for non-glycosylated and glycosylated Im7 respectively.

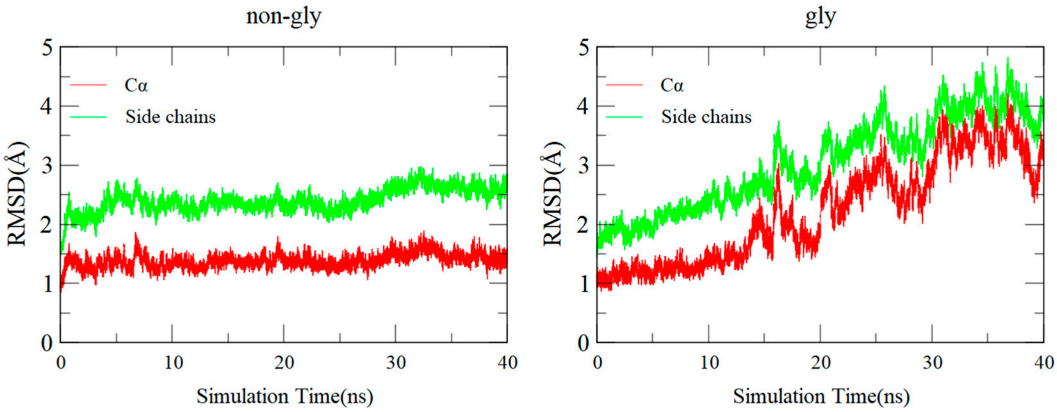

Figure S4. Variation in RMSD based on Cα and sidechain atoms for non-glycosylated and glycosylated Im7.

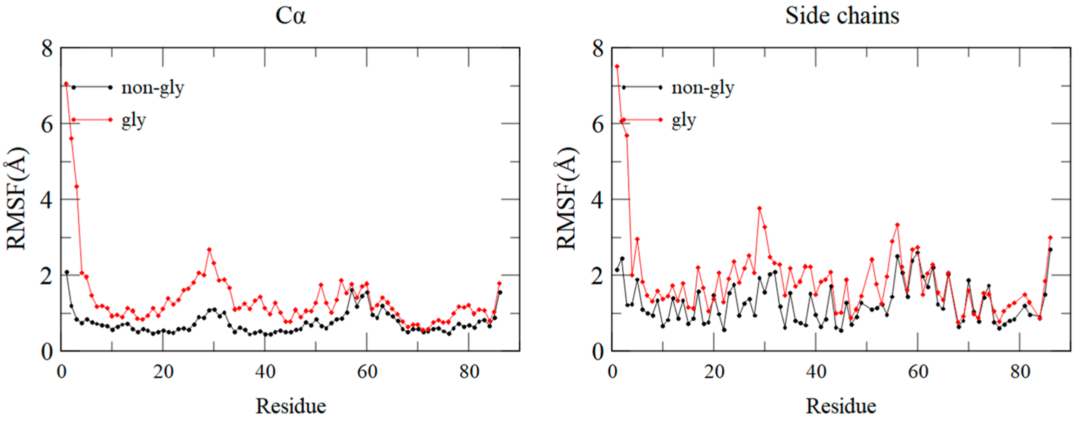

Figure S5. Cα-RMSF and sidechain-RMSF for non-glycosylated and glycosylated Im7.
